# Left atrial area index provides the best prediction of atrial fibrillation in ischemic stroke patients: results from the LAETITIA observational study

**DOI:** 10.3389/fneur.2023.1237550

**Published:** 2023-09-27

**Authors:** Angelika Alonso, Josephine Kraus, Anne Ebert, Valeriya Nikolayenko, Mathieu Kruska, Vesile Sandikci, Hendrik Lesch, Daniel Duerschmied, Michael Platten, Stefan Baumann, Kristina Szabo, Ibrahim Akin, Christian Fastner

**Affiliations:** ^1^Department of Neurology, University Medical Centre Mannheim and Mannheim Centre for Translational Neurosciences, Medical Faculty Mannheim, Heidelberg University, Mannheim, Germany; ^2^Medical Faculty Mannheim, Heidelberg University, Mannheim, Germany; ^3^Department of Cardiology, Angiology, Haemostaseology and Medical Intensive Care, University Medical Centre Mannheim, Medical Faculty Mannheim, Heidelberg University, European Center for AngioScience (ECAS), German Center for Cardiovascular Research (DZHK) Partner Site Heidelberg/Mannheim, Mannheim, Germany; ^4^Department of Geriatrics, University Medical Centre Mannheim, Medical Faculty Mannheim, Heidelberg University, Mannheim, Germany

**Keywords:** left atrial size, left atrial enlargement, atrial cardiomyopathy, thromboembolism, stroke workup, echocardiography

## Abstract

**Background and aims:**

Left atrial (LA) enlargement has been repeatedly shown to be associated with the diagnosis of atrial fibrillation (AF). In clinical practice, several parameters are available to determine LA enlargement: LA diameter index (LADI), LA area index (LAAI), or LA volume index (LAVI). We investigated the predictive power of these individual LA parameters for AF in patients with acute ischemic stroke or transient ischemic attack (TIA).

**Methods:**

LAETITIA is a retrospective observational study that reflects the clinical reality of acute stroke care in Germany. Consecutive patient cases with acute ischemic cerebrovascular event (CVE) in 2019 and 2020 were identified from the Mannheim stroke database. Predictive power of each LA parameter was determined by the area under the curve (AUC) of receiver operating characteristic curves. A cutoff value was determined. A multiple logistic regression analysis was performed to confirm the strongest LA parameter as an independent predictor of AF in patients with acute ischemic CVE.

**Results:**

A total of 1,910 patient cases were included. In all, 82.0% of patients had suffered a stroke and 18.0% had a TIA. Patients presented with a distinct cardiovascular risk profile (reflected by a CHA_2_DS_2_-VASc score ≥2 prior to hospital admission in 85.3% of patients) and were moderately affected on admission [median NIHSS score 3 (1; 8)]. In total, 19.5% of patients had pre-existing AF, and 8.0% were newly diagnosed with AF. LAAI had the greatest AUC of 0.748, LADI of 0.706, and LAVI of 0.719 (each *p* < 0.001 vs. diagonal line; AUC-LAAI vs. AUC-LADI *p* = 0.030, AUC-LAAI vs. AUC-LAVI *p* = 0.004). LAAI, increasing NIHSS score on admission, and systolic heart failure were identified as independent predictors of AF in patients with acute ischemic CVE. To achieve a clinically relevant specificity of 70%, a cutoff value of ≥10.3 cm^2^/m^2^ was determined for LAAI (sensitivity of 69.8%).

**Conclusion:**

LAAI revealed the best prediction of AF in patients with acute ischemic CVE and was confirmed as an independent risk factor. An LAAI cutoff value of 10.3 cm^2^/m^2^ could serve as an inclusion criterion for intensified AF screening in patients with embolic stroke of undetermined source in subsequent studies.

## Introduction

Atrial fibrillation (AF)-related cardiac embolism causes more severe strokes than any other etiology (1). Although the overall incidence of stroke has decreased (2), the number of cardioembolic ischemic strokes has tripled during the past few decades (3). This is mainly due to an aging society with a higher life expectancy, which is more susceptible to AF and AF-related complications (4).

Identifying patients with AF is critical to preventing recurrent stroke as thromboembolic risk can be well controlled with the administration of oral anticoagulation (OAC) (4). AF is easily diagnosed on a surface electrocardiogram (ECG). However, capturing this arrhythmia on an ECG is much more difficult as it is mainly paroxysmal in nature and thus does not necessarily occur during the post-stroke surveillance period (5). Therefore, it is important to confidently identify stroke patients with increased risk for silent paroxysmal AF and subject them to more intensive monitoring. Atrial cardiomyopathy (ACM), defined by structural (i.e., left atrial (LA) fibrosis and enlargement) and electrophysiological (i.e., scarred areas with conduction delays, molecular remodeling, and atrial ectopy) changes, is the driving factor for the development of AF (6). LA enlargement is an easily detectable marker of ACM that is simple to use in routine echocardiography (as opposed to more sophisticated methods such as cardiac magnetic resonance imaging (MRI) or electrophysiological studies) (6). A significant association between LA enlargement and AF detection after embolic stroke of undetermined source (ESUS), all-type stroke, stroke recurrence, and covert brain infarcts has been proven in prior studies (7–9). Those studies also revealed that LA diameter and LA volume are excellent independent predictors of cardioembolic stroke, even in patients without AF (10). Most importantly, LA enlargement can be used to predict AF (4).

The vast majority of these studies have used the LA end-systolic volume index (LAVI; indexed to the patient's body surface area) as a surrogate for LA enlargement. However, following current clinical practice, besides LAVI, the LA end-systolic diameter index (LADI) and the LA end-systolic area index (LAAI) are also routinely measured at most centers. The optimal LA parameter for detecting AF in stroke patients remains the subject of ongoing debate. To the best of our knowledge, data from the Mannheim LA DimEnsions in STroke wIth ATrial FIbrillAtion (LAETITIA) observational study are the first to address the question of which of these three LA parameters offers the best prediction of AF in patients with acute ischemic cerebrovascular events (CVE) when compared in one real-world patient population. For this LA parameter, we intended to define a cutoff value that might be implemented to identify the appropriate candidates for intensified AF screening in consecutive prospective studies.

## Methods

### Study population and data collection

In a retrospective approach, we identified all patients from the prospectively collected stroke database who were treated for acute ischemic stroke or transient ischemic attack (TIA) at the stroke unit of the University Medical Centre Mannheim, Germany, between January 2019 and December 2020. Patients received etiological stroke evaluation, which included laboratory testing, neuroimaging, i.e., computed tomography (CT) or MRI of the neurocranium, 12-lead ECG on admission, transthoracic echocardiography, and cardiac telemetry monitoring for at least 24 h in TIA patients and at least 48 h in patients with acute ischemic stroke. The presence of AF was defined according to the criteria of the 2020 European Society of Cardiology Guidelines for the Diagnosis and Management of Atrial Fibrillation (4). The stroke database had received a positive ethics vote (2013-813R-MA) from the Ethics Committee II of the Medical Faculty Mannheim, Heidelberg University, Mannheim, Germany.

In addition to the baseline characteristics and pre-existing conditions, the following data were obtained from the stroke database: functional scores on admission and at hospital discharge (i.e., National Institutes of Health Stroke Scale (NIHSS) score and modified Rankin Scale score), the CHA_2_DS_2_-VASc score prior to admission and at hospital discharge, medication prior to admission and at hospital discharge, as well as laboratory values on admission. Systolic heart failure was defined as the presence of a left ventricular ejection fraction (LVEF) ≤ 40% plus clinical signs of heart failure (11).

To classify the stroke etiology, we used a modification of the categories proposed by Amarenco et al. (12), i.e., small vessel disease, atherosclerosis, cardiac source, ESUS, and other unidentified causes. Details of neuroimaging and information about therapeutic interventions were collected.

The echocardiographic examinations were performed by specialized physicians of the Department of Cardiology of the University Medical Centre Mannheim, Germany. The LA parameters were determined according to the recommendations of the European Association of Cardiovascular Imaging, whereby data were only collected if the individual anatomy permitted sufficient echocardiographic quality for the respective parameter. LADI was assessed by two-dimensional echocardiography, or M-mode, in the parasternal long axis. LAAI or LAVI data were collected at a minimum from the apical four-chamber view; whenever possible, data were also collected from the apical two-chamber view, and the mean of both values was used (Supplementary Figure 1).

### Statistical analyses

Statistical analysis was performed with IBM SPSS Statistics version 27 (Armonk, NY, USA) and R Project for Statistical Computing (The R Foundation for Statistical Computing, Vienna, Austria). Continuous variables were expressed as medians and interquartile range (IQR), if not normally distributed; ordinal variables as medians and IQR; and categorical variables as absolute numbers and percentages. Group differences were tested with the chi-square test (or Fisher's exact test in cases of low counts) or the Mann–Whitney *U*-test, respectively. Receiver operating characteristic (ROC) curves were performed to determine the predictive value of LADI, LAAI, or LAVI in relation to AF (for any diagnosis of AF or only for patients with a new diagnosis). This was done exclusively among those patients for whom all three LA parameters had been measured. Cutoff values were determined based on the ROC analyses. The significance of an area under the curve (AUC) vs. a diagonal was calculated based on the Mann–Whitney *U*-test and the DeLong test was used to compare different AUCs. We further performed a multiple logistic regression model with forward selection (likelihood ratio) and AF as the dependent variable, with LVEF ≤ 40%, LV diastolic dysfunction, severe mitral regurgitation, CHA_2_DS_2_-VASc score ≥2, coronary artery disease (CAD), NIHSS score on admission, and LAAI as predictors. *P*-values < 0.05 were considered statistically significant. These statistics are based on the available cases.

## Results

### Patient characteristics

A total of 1,910 patient cases were included [median age 75 (64; 83) years, 45% women; Table 1], of whom, 27.5% of patients suffered from AF (pre-existing or new diagnosis). There was only a small gap (1.9%) between the percentage of patients with pre-existing AF and that of patients on OAC. In addition, 85.3% of patients had a CHA_2_DS_2_-VASc-score ≥2 prior to admission. This was accompanied by more than three-quarters of patients with arterial hypertension, approximately one-quarter with diabetes mellitus, and approximately one-fifth with pre-existing CAD. The median NIHSS score on admission was 3 (IQR 1, 8).

**Table 1:** Patient characteristics of the LAETITIA population.

**Total number of patients, *n***	**1,910**
Female patients, *n* (%)	861 (45.1)
Age [years], median (IQR)	75 (64; 83)
Body mass index [kg/m^2^], median (IQR)	26.1 (23.7; 29.4)
**Data on cerebral lesion**	
Cerebrovascular event, each *n* (%)	
•Acute ischemic stroke	1,566 (82.0)
•Transient ischemic attack	344 (18.0)
First event of cerebral ischemia, *n* (%)	1,486 (77.8)
Stroke etiology, each *n* (%)	
•Cardioembolism	628 (32.9)
•Small vessel occlusion	321 (16.8)
•Large artery atherosclerosis	255 (13.4)
•Other determined etiology	48 (2.5)
•Undetermined	658 (34.4)
° ESUS	489 (25.6)
Intracranial vessel occlusion, each *n* (%)	
•Large vessel^1^	198 (10.4)
•Medium vessel^2^	43 (2.3)
Recanalization therapy, each *n* (%)	
•Systemic thrombolysis	454 (23.8)
•Mechanical thrombectomy	223 (11.7)
NIHSS score, each median (IQR)	
•On admission	3 (1; 8)
•At hospital discharge	1 (0; 4)
mRS score, each median (IQR)	
•On admission	3 (1; 4)
•At hospital discharge	2 (0; 4)
**Concomitant diseases**	
Arterial hypertension, each *n* (%)	1,586 (83.0)
•New diagnosis	41 (2.1)
Atrial fibrillation, each *n* (%)	526 (27.5)
•New diagnosis	153 (8.0)
Coronary artery disease, each *n* (%)	424 (22.2)
•New diagnosis	32 (1.7)
Diabetes mellitus, each *n* (%)	556 (29.1)
•New diagnosis	35 (1.8)
Dyslipidemia, *n* (%)	1,314 (69.6)
Advanced chronic kidney disease, *n* (%)	136 (7.1)
Smoker, each *n* (%)	265 (13.9)
•Formerly	88 (4.6)
Systolic heart failure, *n* (%)	116 (6.1)
Previous clinically silent territorial ischemia, *n* (%)	264 (13.8)
CHA_2_DS_2_-VASc score, each median (IQR)	
•Prior to admission	4 (2; 5)
•At hospital discharge	6 (4; 6)
History of bleeding, *n* (%)	135 (7.1)
**Medication** ^3^	
Oral anticoagulation, each *n* (%)	
•Prior to admission	332 (17.6)
•At hospital discharge	455 (25.0)
Antiplatelet agent, each *n* (%)	
•Prior to admission	600 (32.6)
•At hospital discharge	1,361 (74.7)
Antihypertensive drug, each *n* (%)	
•Prior to admission	1,339 (71.4)
•At hospital discharge	1,478 (81.2)
Lipid lowering drug, each *n* (%)	
•Prior to admission	627 (34.3)
•At hospital discharge	1,778 (97.5)
**Laboratory values on admission**	
Potassium [mmol/L], median (IQR)	4.0 (3.7; 4.3)
Creatinine [mg/dl], median (IQR)	1.0 (0.8; 1.3)
eGFR [ml/min/1.73 m^2^], median (IQR)	58 (45; 75)
HbA1c [%], median (IQR)	5.8 (5.4; 6.5)

^1^Intracranial internal carotid artery, M1 or M2 of middle cerebral artery, basilar artery; ^2^M3 or M4 of middle cerebral artery, A1 or A2 of anterior cerebral artery, P1-P3 of posterior cerebral artery; ^3^more than one substance of each class could be used per patient. eGFR, estimated glomerular filtration rate; ESUS, embolic stroke of undetermined source; IQR, interquartile range; mRS, modified Rankin Scale; NIHSS, National Institutes of Health Stroke Scale.

In addition, 82.0% of patients had suffered an acute ischemic stroke, while 18.0% presented with a TIA. In 77.8% of the cases, it was the first acute ischemic CVE. In 13.8% of the patients, brain imaging showed a pre-existing clinically silent territorial ischemia. Analyzing the CVE's etiology, a presumed cardioembolic cause was found in 32.9%, an ESUS in 25.6% of cases, and a small vessel occlusion in 16.8% of cases. In a total of 34.4% of the cases, the etiology remained unclear (embolic or non-embolic infarct pattern). Approximately one-third of the patients received intravenous thrombolysis and/or mechanical thrombectomy.

The group of patients with AF was older and had higher frequencies of cardiovascular disease, including lower biventricular function and larger LAs (Supplementary Table 1).

### Echocardiographic findings

Transthoracic echocardiography was performed in nearly 90% of cases. LVEF was not impaired in the vast majority of patients (83.6%), with LV diastolic dysfunction in 85.4% of patients (Table 2). All LA parameters were determined in approximately one-half of the cases (*n* = 846). Median LADI was 20 (18; 23) (*n* = 935), median LAAI 9.5 (7.7; 11.8) (*n* = 1,069), and median LAVI 23 (17; 33) (*n* = 966), respectively.

**Table 2:** Echocardiographic findings.

**LADI [mm/m^2^], median (IQR)**	**20 (18; 23)**
LAAI [cm^2^/m^2^], median (IQR)	9.5 (7.7; 11.8)
LAVI [cm^3^/m^2^], median (IQR)	23 (17; 33)
LVEF [%], median (IQR)	55 (48; 60)
LV diastolic dysfunction, *n* (%)	1,161 (85.4)
Mitral valve regurgitation grade II/III, *n* (%)	188 (11.4)
Mitral valve stenosis grade II/III, *n* (%)	4 (0.2)
Combined mitral valve vitium grade II/III, *n* (%)	2 (0.1)
Aortic valve regurgitation grade II/III, *n* (%)	66 (4.0)
Aortic valve stenosis grade II/III, *n* (%)	122 (7.4)
Combined aortic valve vitium grade II/III, *n* (%)	10 (0.6)
Bicuspid aortic valve, *n* (%)	9 (0.5)
TAPSE [mm], median (IQR)	21 (19; 25)
Systolic PAP [mmHg], median (IQR)	30 (24; 38)

LAAI, left atrial area index; LADI, left atrial diameter index; LAVI, left atrial volume index; LV (EF), left ventricular (ejection fraction); PAP, pulmonary artery pressure; TAPSE, tricuspid annular plane systolic excursion.

### ROC analyses

**Figure 1:**
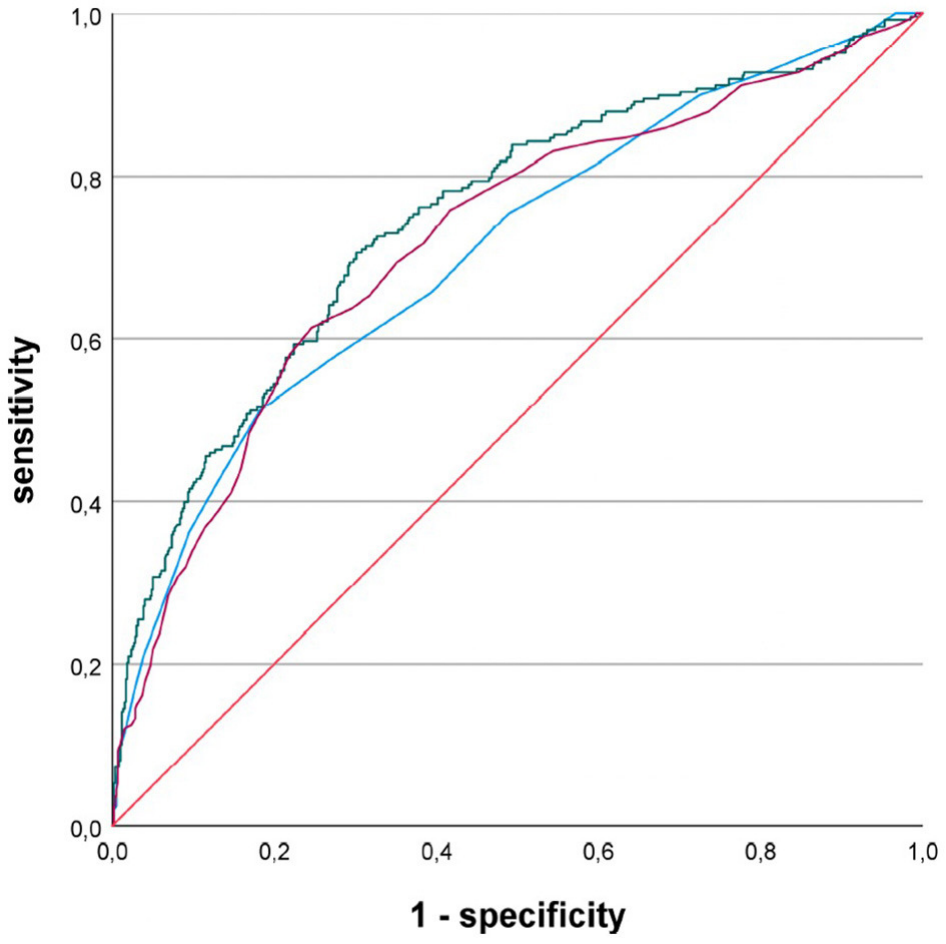
Receiver operating characteristic curves for the performance of different left atrial parameters predicting atrial fibrillation in patients with acute ischemic cerebrovascular event; blue line, left atrial diameter index; green line, left atrial area index; purple line, left atrial volume index.

**Figure 2:**
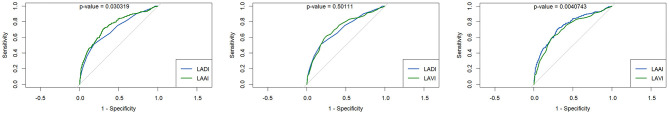
Receiver operating characteristic curves for the performance of different left atrial parameters predicting atrial fibrillation in patients with acute ischemic cerebrovascular event compared by the DeLong test; **(left)** left atrial (LA) diameter index (LADI) vs. LA area index (LAAI), **(middle)** LADI vs. LA volume index (LAVI), **(right)** LAAI vs. LAVI.

When comparing the sensitivity and specificity of the three echocardiographic LA parameters (i.e., LADI, LAAI, or LAVI) in patients with pre-existing or first diagnosed AF, LAAI yielded the greatest AUC: LADI = 0.706, LAAI = 0.748, and LAVI = 0.719, respectively (Figure 1). The AUC of each of these LA parameters reached statistical significance against the diagonal line (each *p* < 0.001). In addition, the AUC of LAAI was statistically significantly larger than that of any other LA parameter (Figure 2; each *p* < 0.05). With a minimum specificity of 70.0%, LAAI was found to be ≥10.3 cm^2^/m^2^ (sensitivity of 69.8%).

**Figure 3:**
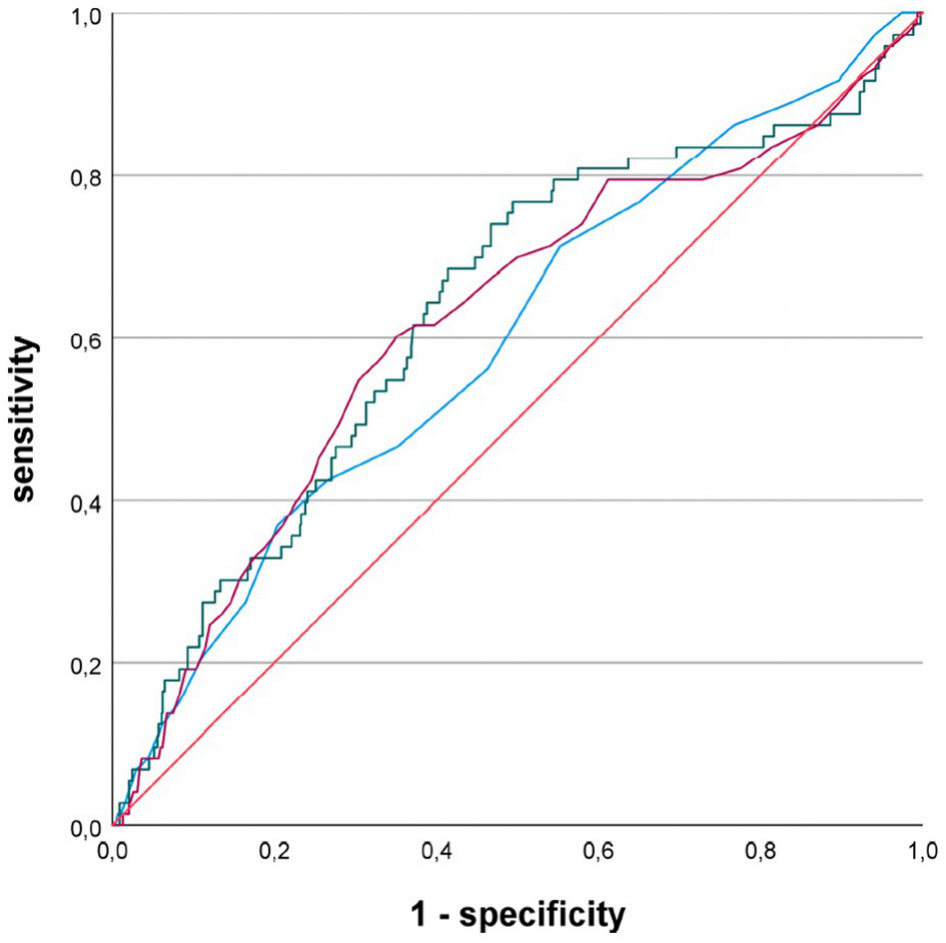
Receiver operating characteristic curves for the performance of different left atrial parameters predicting newly diagnosed atrial fibrillation in patients with acute ischemic cerebrovascular event; blue line, left atrial diameter index; green line, left atrial area index; purple line, left atrial volume index.

In a subgroup of 65 patients with first-diagnosed AF and a presumed cardioembolic stroke etiology, defined as stroke with an embolic infarct pattern and exclusion of large-artery disease, ROC analysis again yielded the greatest AUC for LAAI: LADI = 0.597, LAAI = 0.632, LAVI = 0.619 (Figure 3). Again, all three LA parameters reached statistical significance comparing their AUC against the diagonal line (LADI: *p* = 0.006, LAAI: *p* < 0.001, and LAVI: *p* = 0.0001, respectively). In this limited subgroup, there was no statistically significant difference between the AUCs of the LA parameters using the DeLong test. With a minimum specificity of 70.0%, LAAI was found to be ≥11.2 cm^2^/m^2^ (sensitivity of 47.9%).

### Multiple logistic regression analysis

A multiple logistic regression model with forward selection for predicting AF in acute ischemic CVE patients was performed. Within three steps, the presence of systolic heart failure (*p* < 0.001; odds ratio (OR) 2.60 [1.68–4.04]), NIHSS score on admission (*p* < 0.001, OR 1.05 [1.02–1.09]), and LAAI (*p* < 0.001, 1.31 [1.24–1.40]) remained significant predictors in the model (Table 3). Meanwhile, severe mitral valve regurgitation, LV diastolic dysfunction, CHA_2_DS_2_-VASc score ≥2 prior to admission, and CAD did not add significant predictions to the model and were removed (each p > 0.05).

**Table 3:** Predictors of atrial fibrillation in patients with acute ischemic cerebrovascular event.

**Predictor**	***p*-value**	**OR [CI]**
**Variables removed**		
Mitral valve regurgitation	0.37	-
LV diastolic dysfunction	0.58	-
CHA_2_DS_2_-VASc score ≥2 prior to admission	0.18	-
Coronary artery disease	0.07	-
**Variables selected**		
Reduced LVEF ≤ 40%	<0.001	2.60 [1.68–4.04]
Increasing NIHSS score on admission	<0.001	1.05 [1.02–1.09]
Increasing LAAI	<0.001	1.31 [1.24–1.40]

*P*-values ≤ 0.05 are considered statistically significant; CI, confidence interval; LAAI, left atrial area index; LV (EF), left ventricular (ejection fraction); NIHSS, National Institutes of Health Stroke Scale; OR, odds ratio.

## Discussion

LAAI yielded the best prediction for AF among patients with acute ischemic CVE. Assuming a clinically relevant specificity of at least 70%, LAAI ≥10.3 cm^2^/m^2^ was indicative of the presence of AF in this patient group. This cutoff value could thus serve as a basis for further studies aiming at intensified screening for AF in acute ischemic stroke patients.

The value of the various parameters defining LA enlargement is controversial, although most recent outcome studies have been performed with 2D echocardiographic LAVI (13–15). In the vast majority of cases, LAVI has been compared against LADI, which is typically measured using M-mode echocardiography. The latter is in fact poorly predictive of actual LA geometry, as LA dilatation occurs primarily in the longitudinal direction rather than the anterior-posterior direction (16). However, LA volume can only be determined indirectly by extrapolation (17), which limits the exactness and the intra- or interobserver accuracy (18). These extrapolations are based on assumptions about LA geometry, which do not take into account the high complexity of the LA in ACM (6). In this respect, it is not surprising that the repeatedly propagated LA volume measurement for the determination of LA enlargement in echocardiography does not show a good correlation with the actual LA volume measured by CT (18, 19). The LA area, on the other hand, is easy to assess and does not require any extrapolation in the third dimension (17). However, it requires correct angulation that considers an axis different from that of the LV (18). It can be assumed that the good reproducibility of the LAAI contributes to its higher predictive value. Indexed LA parameters are better correlated with the patient's stature, allowing the elimination of this possible confounder inherent in absolute, non-indexed values (17).

The predictive value of LAAI for the detection of AF in patients with acute ischemic CVE is underlined by the fact that this LA parameter also had the highest AUC in a subgroup of patients with newly diagnosed AF, although results were more heterogeneous due to the small group size. In these patients, it may be assumed that the first diagnosis was associated with less advanced ACM (6). Such patients usually show less structural remodeling (6), so a sensitivity of only 48% could be achieved to reach a minimum specificity of 70%. Moreover, these values were certainly negatively influenced by the small group size as well.

The study sample was highly representative of a patient group with acute ischemic CVE and had numerous risk factors for LA enlargement. These risk factors, typically consisting of LV diastolic dysfunction and higher grade mitral regurgitation, were subjected to multiple logistic regression analysis together with CAD, systolic heart failure, CHA_2_DS_2_-VASc score ≥2, increasing NIHSS score on admission, and LAAI. Interestingly, no significant relation was seen for the CHA_2_DS_2_-VASc score depicting global cardiovascular risk. Only systolic heart failure, the NIHSS score, and LAAI, i.e., an upstream condition of the individual risk factors, were predictive of AF in patients with acute ischemic CVE. Systolic heart failure has been repeatedly described as a major cause of AF and ACM (6), and this relation was also shown for patients with acute ischemic CVE in the present analysis. An increasing degree of disability on admission is associated with higher stroke severity, as has been well described for AF-related stroke (1). The fact that LAAI emerged as an independent predictor over single cardiovascular risk factors in this multiple logistic regression analysis further strengthens its clinical significance.

The clinical significance of recognizing an increase in LAAI as an accurate and reliably detectable predictor arises from its potential implications for screening individuals with suspected silent AF. A relevant number of acute ischemic strokes are due to AF without detection during telemetric monitoring in the stroke unit (5). So far, there is no consensus on how to further evaluate stroke patients for silent AF after hospital discharge (20, 21). A sensitive but also invasive and expensive method of monitoring is an implantable loop recorder (22). Due to these limitations, this diagnostic tool is not widely utilized, and there are currently no standardized algorithms for its application in the follow-up of suspected silent AF. Patients with the highest probability of AF detection need to be identified to justify the use of this intensive form of monitoring over an external 24-h ECG (4). The American Heart Association/American Stroke Association and European Stroke Organisation guidelines for the Early Management of Acute Ischemic Stroke recommend using transthoracic echocardiography to further guide secondary prevention of recurrent stroke in unclear cases (20, 21). Thorough cardiac imaging can reveal competing causes, such as a persistent foramen ovale (23). In this context, a low LAAI should not serve to exclude ESUS patients from serial 24-h ECGs, as required by the relevant guidelines. Instead, based on the presented hypothesis-generating data, we consider increased LAAI ≥10.3 cm^2^/m^2^ as a good predictor for the selection of even intensified screening for silent AF after acute ischemic stroke with embolic infarct pattern (e.g., by an implantable loop recorder). Multilevel algorithms that consider other clinical predictors in addition to LA enlargement indicated by LAAI as an entry requirement, as summarized in scores such as the C_2_HEST score (24), could be used to identify patients who benefit most from intensified screening by implantation of a loop recorder.

In no way should it be inferred that LA enlargement, as measured by an increase in LAAI, can be used to infer the presence of AF or the immediate need for OAC therapy. Two international randomized trials that aimed to demonstrate that the use of non-vitamin K antagonist OAC is beneficial in patients with large atria even in the absence of clinical AF failed with this hypothesis (25, 26). Indeed, ACM is more than a mere structural enlargement but is also associated with electrical changes in the atrial myocardium (6). Thus, the aim should be to index, by good preselection, those patients in whom intensified monitoring can additionally identify the electrical component of ACM, i.e., AF, in the course.

It should be emphasized that LAAI is easy to determine on almost all echo devices, thus ensuring its broad implementation in clinical practice. The clinical success of such a strategy should be further evaluated in larger prospective and randomized studies.

### Limitations

This prospectively compiled observational study with retrospectively selected cases has the inherent limitations of such a data collection. Regarding the echocardiographic data, no core lab was used, but image data collection was performed by recognized experts in the field of echocardiography. However, despite a high level of expertise, image acquisition is limited in certain patients (e.g., due to obesity or paresis-related position restrictions). In this respect, it is not surprising that the patients from the LAETITIA patient population, in whom LA parameters could not be collected completely, tended to have a higher body mass index and were more severely affected by stroke (Supplementary Table 2). However, the absolute differences in baseline characteristics were small, and there was only a mild effect size in a large group of patients. Data derived from LAETITIA aim to reflect clinical reality. That is why we also consciously show limitations, and our results are transferable to those patients in whom LA parameters can be practically collected during stroke workup. External validation of the cutoff value has not been performed to date. Rather, we would like to provide the results of our large retrospective study aggregating comprehensive data from stroke care as a basis for prospective validation studies.

## Conclusion

Of all the available echocardiographic LA parameters in clinical practice, LAAI revealed the best prediction of AF in patients with acute ischemic CVE. We identified a cutoff value of 10.3 cm^2^/m^2^ that resulted in a clinically relevant specificity of at least 70%. We propagate using this LAAI cutoff value to trigger intensified screening for silent AF in acute ischemic CVE patients with an embolic infarct pattern as a basis for prospective randomized trials.

## Data availability statement

According to the General Data Protection Regulation, the original data may not be published. All data relevant for the interpretation of the results were provided in aggregated form in the article and tables. Requests to access these datasets should be directed to christian.fastner@umm.de.

## Ethics statement

The studies involving humans were approved by the Ethics Committee II of the Medical Faculty Mannheim, Heidelberg University, Mannheim, Germany. The studies were conducted in accordance with the local legislation and institutional requirements. Written informed consent for participation was not required from the participants or the participants' legal guardians/next of kin in accordance with the national legislation and institutional requirements.

## Author contributions

The study was designed by AA and CF, with detailed elaboration by JK. AA, MP, and KS are responsible for the data content of the underlying stroke database. Specific data collection was led by AA, JK, VN, and CF. Statistical data analysis was led by AE. AA, JK, and CF wrote the first draft of the manuscript. MK, HL, DD, MP, SB, KS, and IA contributed to contextualizing the study results. VS supported the statistical analyses required during the revision process. All authors reviewed and edited the manuscript and approved the final version of the manuscript.
