# Supplementary material for: Left atrial area index provides the best prediction of atrial fibrillation in ischemic stroke patients: results from the LAETITIA observational study
Source: Front Neurol. 2023 Sep 27;14:1237550. doi: 10.3389/fneur.2023.1237550 (PMC10580428; doi:10.3389/fneur.2023.1237550)
Supplement: Supplementary file 1 [file Table_1.pdf]

Supplementary Table 1: Group comparison of patients with (+) atrial fibrillation (AF) versus those without (-)

	AF +	AF -	*p*-value	*OR or r*
Total number of patients, n	526	1384		
Female patients, n (%)	268 (51.0)	593 (42.8)	**0.001**	1.386
Age [years], median (IQR)	82 (75; 86)	71 (60; 81)	**< 0.001**	-0.344
Body mass index [kg/m^2], median (IQR)	26.0 (23.5; 29.4)	26.2 (23.9; 29.4)	0.31	-0.029
Data on cerebral lesion				
Cerebrovascular event, each n (%)			**< 0.001**	0.596
• acute ischemic stroke	458 (87.1)	1108 (80.1)		
• transient ischemic attack	68 (12.9)	276 (19.9)		
First event of cerebral ischemia, n (%)	386 (73.4)	1100 (79.5)	**0.004**	1.405
Stroke etiology, each n (%)				
• cardioembolism	449 (85.4)	179 (12.9)	**< 0.001**	39.255
• small vessel occlusion	29 (5.5)	292 (21.1)	**< 0.001**	0.218
• large artery atherosclerosis	34 (6.5)	221 (16.0)	**< 0.001**	0.364
• other determined etiology	5 (1.0)	43 (3.1)	**0.005**	0.299
• undetermined	9 (1.7)	649 (46.9)	**< 0.001**	0.020
○ ESUS	7 (1.3)	482 (34.8)	**< 0.001**	0.025
Intracranial vessel occlusion, each n (%)				
• large vessel[1]	100 (19.0)	98 (7.1)	**< 0.001**	3.080
• medium vessel[2]	14 (2.7)	29 (2.1)	0.46	1.278
Recanalization therapy, each n (%)				
• systemic thrombolysis	121 (23.0)	333 (24.1)	0.628	0.943
• mechanical thrombectomy	100 (19.0)	123 (8.9)	**< 0.001**	2.407
NIHSS score, each median (IQR)				
• on admission	6 (2; 14)	3 (1; 6)	**< 0.001**	-0.214
• at hospital discharge	3 (1; 8)	1 (0; 3)	**< 0.001**	-0.205
mRS score, each median (IQR)				
• on admission	4 (2; 5)	2 (1; 4)	**< 0.001**	-0.237
• at hospital discharge	3 (1; 5)	1 (0; 3)	**< 0.001**	-0.245
Concomitant diseases				
Arterial hypertension, each n (%)	473 (89.9)	1113 (80.4)	**< 0.001**	2.173
• new diagnosis	4 (0.8)	37 (2.7)	**0.008**	0.279
Coronary artery disease, each n (%)	168 (31.9)	256 (18.5)	**< 0.001**	2.068
• new diagnosis	10 (1.9)	22 (1.6)	0.64	1.200

Diabetes mellitus, each n (%)<br>• new diagnosis	152 (28.9)<br>7 (1.3)	404 (29.2)<br>28 (2.0)	0.90<br>0.44	0.986<br>0.653
Dyslipidemia, n (%)	335 (64.7)	979 (71.5)	**0.004**	1.371
Advanced chronic kidney disease, n (%)	63 (12.0)	73 (5.3)	**< 0.001**	0.410
Smoker, each n (%)<br>• formerly	32 (6.1)<br>28 (5.3)	233 (16.8)<br>60 (4.3)	**< 0.001**<br>0.36	0.320<br>1.240
Systolic heart failure, n (%)	68 (13.5)	48 (3.5)	**< 0.001**	0.233
Small vessel disease, Fazekas classification, each n (%)<br>• 0<br>• 1<br>• 2<br>• 3	47 (8.9)<br>180 (34.2)<br>148 (28.1)<br>151 (28.7)	308 (22.3)<br>542 (39.2)<br>266 (19.2)<br>268 (19.4)	**< 0.001**<br>**0.047**<br>**< 0.001**<br>**< 0.001**	0.343<br>0.808<br>1.646<br>1.677
Previous clinically silent territorial ischemia, n (%)	74 (14.1)	190 (13.7)	0.86	0.974
CHA_2DS_2-VASc score, each median (IQR)<br>• prior to admission<br>• at hospital discharge	4 (4; 5)<br>6 (5; 7)	3 (2; 5)<br>5 (4; 6)	**< 0.001**<br>**< 0.001**	-0.263<br>-0.286
History of bleeding, n (%)	62 (11.8)	73 (5.3)	**< 0.001**	0.417
<u>Medication[3]</u>				
Oral anticoagulation, each n (%)<br>• prior to admission<br>• at hospital discharge	271 (52.3)<br>390 (80.7)	1311 (4.4)<br>65 (4.9)	**< 0.001**<br>**< 0.001**	23.580<br>82.194
Antiplatelet agent, each n (%)<br>• prior to admission<br>• at hospital discharge	115 (22.9)<br>103 (21.3)	485 (36.6)<br>1258 (94.0)	**< 0.001**<br>**< 0.001**	0.522<br>0.017
Antihypertensive drug, each n (%)<br>• prior to admission<br>• at hospital discharge	436 (85.3)<br>450 (93.4)	903 (66.2)<br>1028 (76.8)	**< 0.001**<br>**< 0.001**	2.968<br>4.241
Lipid lowering drug, each n (%)<br>• prior to admission<br>• at hospital discharge	202 (40.6)<br>468 (96.9)	425 (31.9)<br>1310 (97.8)	**< 0.001**<br>0.29	1.461<br>0.715
<u>Laboratory values on admission</u>				
Potassium [mmol/l], median (IQR)	4.0 (3.7; 4.4)	3.9 (3.7; 4.3)	**0.027**	-0.051
Creatinine [mg/dl], median (IQR)	1.1 (0.9; 1.4)	1.0 (0.8; 1.2)	**< 0.001**	-0.158
eGFR [ml/min/1,73 m^2], median (IQR)	48 (37; 59)	62 (48; 81)	**< 0.001**	-0.309
HbA1c [%], median (IQR)	5.9 (5.5; 6.5)	5.8 (5.4; 6.4)	0.13	-0.036

Echocardiographic findings				
LADI [mm/m^2], median (IQR)	22 (20; 26)	19 (17; 22)	**< 0.001**	-0.317
LAAI [cm^2/m^2], median (IQR)	11.7 (9.5; 14.2)	8.9 (7.4; 10.9)	**< 0.001**	-0.369
LAVI [cm^3/m^2], median (IQR)	32 (23; 43)	21 (16; 29)	**< 0.001**	-0.341
LVEF [%], median (IQR)	51 (40; 58)	56 (50; 61)	**< 0.001**	-0.284
LV diastolic dysfunction, n (%)	254 (91.4)	907 (83.3)	**0.002**	2.042
Mitral valve regurgitation grade II/III, n (%)	116 (27.7)	72 (5.9)	**< 0.001**	6.125
Mitral valve stenosis grade II/III, n (%)	1 (0.2)	3 (0.2)	1.00	0.974
Combined mitral valve vitium grade II/III, n (%)	2 (0.5)	0 (0)	0.065	---
Aortic valve regurgitation grade II/III, n (%)	19 (4.5)	47 (3.8)	0.54	1.186
Aortic valve stenosis grade II/III, n (%)	59 (14.0)	63 (5.2)	**< 0.001**	3.009
Combined aortic valve vitium grade II/III, n (%)	5 (1.2)	5 (0.4)	0.14	2.935
Bicuspid aortic valve, n (%)	3 (0.7)	6 (0.5)	0.70	0.685
TAPSE [mm], median (IQR)	19 (17; 23)	22 (20; 25)	**< 0.001**	-0.284
Systolic PAP [mmHg], median (IQR)	35 (28; 44)	27 (22; 35)	**< 0.001**	-0.301

[1]intracranial internal carotid artery, M1 or M2 of middle cerebral artery, basilar artery; [2]M3 or M4 of middle cerebral artery, A1 or A2 of anterior cerebral artery, P1-P3 of posterior cerebral artery; [3]more than one substance of each class could be used per patient

eGFR, estimated glomerular filtration rate; ESUS, embolic stroke of undetermined source; IQR, interquartile range; LAAI, left atrial area index; LADI, left atrial diameter index; LAVI, left atrial volume index; LV(EF), left ventricular (ejection fraction); mRS, modified Rankin Scale; NIHSS, National Institutes of Health Stroke Scale; OR, odds ratio; PAP, pulmonary artery pressure; TAPSE, tricuspid annular plane systolic excursion

bold font indicates $p < 0.05$
