# Supplementary material for: Left atrial area index provides the best prediction of atrial fibrillation in ischemic stroke patients: results from the LAETITIA observational study
Source: Front Neurol. 2023 Sep 27;14:1237550. doi: 10.3389/fneur.2023.1237550 (PMC10580428; doi:10.3389/fneur.2023.1237550)
Supplement: Supplementary file 2 [file Table_2.pdf]

Supplementary Table 2: Group comparison of patients with (+) and without (-) left atrial (LA) parameters in echocardiographic assessment

	LA parameters +	LA parameters -	*p*-value	*OR or r*
Total number of patients, n	846	1064		
Female patients, n (%)	379 (44.8)	482 (45.3)	0.83	1.020
Age [years], median (IQR)	76 (68; 83)	74 (61; 83)	**< 0.001**	-0.085
Body mass index [kg/m^2], median (IQR)	26.0 (23.6; 29.2)	26.6 (24.2; 30.1)	**0.010**	-0.085
Data on cerebral lesion				
Cerebrovascular event, each n (%)<br>• acute ischemic stroke<br>• transient ischemic attack	 166 (19.6)<br>680 (80.4)	 178 (16.7)<br>886 (83.3)	0.10	0.823
First event of cerebral ischemia, n (%)	654 (77.3)	832 (78.2)	0.64	0.950
Stroke etiology, each n (%)<br>• cardioembolism<br>• small vessel occlusion<br>• large artery atherosclerosis<br>• other determined etiology<br>• undetermined<br>○ ESUS	 265 (31.3)<br>163 (19.3)<br>110 (13.0)<br>21 (2.5)<br>287 (33.9)<br>213 (25.2)	 363 (34.1)<br>158 (14.8)<br>145 (13.6)<br>27 (2.5)<br>371 (34.9)<br>276 (25.9)	 0.20<br>**0.010**<br>0.69<br>0.94<br>0.67<br>0.70	 1.135<br>0.731<br>1.056<br>1.023<br>1.043<br>1.041
Intracranial vessel occlusion, each n (%)<br>• large vessel[1]<br>• medium vessel[2]	 68 (8.0)<br>19 (2.2)	 130 (12.2)<br>24 (2.3)	 **0.003**<br>0.99	 1.592<br>1.004
Recanalization therapy, each n (%)<br>• systemic thrombolysis<br>• mechanical thrombectomy	 192 (22.7)<br>81 (9.6)	 262 (24.6)<br>142 (13.3)	 0.33<br>**0.011**	 1.113<br>1.455
NIHSS score, each median (IQR)<br>• on admission<br>• at hospital discharge	 3 (1; 6)<br>1 (0; 3)	 4 (1; 10)<br>2 (0; 6)	 **< 0.001**<br>**< 0.001**	 -0.112<br>-0.130
mRS score, each median (IQR)<br>• on admission<br>• at hospital discharge	 3 (1; 4)<br>2 (0; 3)	 3 (1; 5)<br>2 (0; 4)	 **< 0.001**<br>**< 0.001**	 -0.085<br>-0.121
Concomitant diseases				
Arterial hypertension, each n (%)<br>• new diagnosis	727 (85.9)<br>18 (2.1)	859 (80.7)<br>23 (2.2)	**0.003**<br>1.00	0.686<br>1.016
Atrial fibrillation, each n (%)	248 (29.3)	278 (26.1)	0.12	0.853

• new diagnosis	73 (8.6)	80 (7.5)	0.38	0.861
Coronary artery disease, each n (%)<br>• new diagnosis	201 (23.8)<br>11 (1.3)	223 (21.0)<br>21 (2.0)	0.14<br>0.26	0.851<br>1.528
Diabetes mellitus, each n (%)<br>• new diagnosis	270 (31.9)<br>19 (2.2)	286 (26.9)<br>16 (1.5)	**0.016**<br>0.23	0.784<br>0.665
Dyslipidemia, n (%)	597 (70.8)	717 (68.7)	0.32	1.107
Advanced chronic kidney disease, n (%)	60 (7.1)	76 (7.1)	0.96	0.991
Smoker, each n (%)<br>• formerly	109 (12.9)<br>31 (3.7)	156 (14.7)<br>57 (5.4)	0.26<br>0.079	1.163<br>1.490
Systolic heart failure, n (%)	778 (92.3)	973 (95.0)	**0.015**	1.594
Small vessel disease, Fazekas classification, each n (%)<br>• 0<br>• 1<br>• 2<br>• 3	 131 (15.5)<br>347 (41.0)<br>191 (22.6)<br>177 (20.9)	 224 (21.1)<br>375 (35.2)<br>223 (21.0)<br>242 (22.7)	 **0.002**<br>**0.010**<br>0.39<br>0.34	 1.455<br>0.783<br>0.909<br>1.113
Previous clinically silent territorial ischemia, n (%)	117 (13.9)	147 (13.8)	0.98	1.004
CHA_2DS_2-VASc score, each median (IQR)<br>• prior to admission<br>• at hospital discharge	 4 (3; 5)<br>6 (5; 6)	 4 (2; 5)<br>5 (4; 6)	 **< 0.001**<br>**< 0.001**	 -0.095<br>-0.111
History of bleeding, n (%)	62 (7.3)	73 (6.9)	0.70	1.072
Medication[3]				
Oral anticoagulation, each n (%)<br>• prior to admission<br>• at hospital discharge	 159 (18.8)<br>233 (28.0)	 173 (16.5)<br>222 (22.4)	 0.19<br>**0.006**	 0.854<br>0.741
Antiplatelet agent, each n (%)<br>• prior to admission<br>• at hospital discharge	 270 (32.9)<br>603 (72.6)	 330 (32.4)<br>758 (76.5)	 0.83<br>0.055	 0.979<br>1.230
Antihypertensive drug, each n (%)<br>• prior to admission<br>• at hospital discharge	 619 (74.0)<br>701 (84.6)	 720 (69.4)<br>777 (78.4)	 **0.029**<br>**0.001**	 0.797<br>0.663
Lipid lowering drug, each n (%)<br>• prior to admission<br>• at hospital discharge	 291 (35.6)<br>815 (98.1)	 336 (33.2)<br>963 (97.1)	 0.29<br>0.17	 0.901<br>0.652
Laboratory values on admission				
Potassium [mmol/l], median (IQR)	4.0 (3.7; 4.3)	4.0 (3.7; 4.3)	0.30	-0.024
Creatinine [mg/dl], median (IQR)	1.0 (0.8; 1.3)	1.0 (0.8; 1.2)	0.52	-0.015

eGFR [ml/min/1,73 m^2], median (IQR)	56 (44; 71)	59 (45; 79)	0.40	-0.064
HbA1c [%], median (IQR)	5.8 (5.4; 6.4)	5.8 (5.4; 6.5)	0.17	-0.032
Echocardiographic findings				
LVEF [%], median (IQR)	55 (48; 60)	54 (47; 60)	0.23	-0.050
LV diastolic dysfunction, n (%)	587 (83.5)	574 (87.4)	**0.044**	1.367
Mitral valve regurgitation grade II/III, n (%)	121 (14.5)	67 (8.3)	**< 0.001**	0.536
Mitral valve stenosis grade II/III, n (%)	2 (0.2)	2 (0.2)	1.00	1.039
Combined mitral valve vitium grade II/III, n (%)	1 (0.1)	1 (0.1)	1.00	1.039
Aortic valve regurgitation grade II/III, n (%)	39 (4.7)	27 (3.3)	0.18	0.709
Aortic valve stenosis grade II/III, n (%)	74 (8.8)	48 (6.0)	**0.026**	0.653
Combined aortic valve vitium grade II/III, n (%)	6 (0.7)	4 (0.5)	0.75	0.691
Bicuspid aortic valve, n (%)	2 (0.2)	7 (0.9)	0.10	0.274
TAPSE [mm], median (IQR)	21 (19; 25)	21 (19; 25)	0.76	-0.009
Systolic PAP [mmHg], median (IQR)	31 (25; 40)	28 (22; 37)	**0.001**	-0.130

[1]intracranial internal carotid artery, M1 or M2 of middle cerebral artery, basilar artery; [2]M3 or M4 of middle cerebral artery, A1 or A2 of anterior cerebral artery, P1-P3 of posterior cerebral artery; [3]more than one substance of each class could be used per patient

eGFR, estimated glomerular filtration rate; ESUS, embolic stroke of undetermined source; IQR, interquartile range; LAAI, left atrial area index; LADI, left atrial diameter index; LAVI, left atrial volume index; LV(EF), left ventricular (ejection fraction); mRS, modified Rankin Scale; NIHSS, National Institutes of Health Stroke Scale; OR, odds ratio; PAP, pulmonary artery pressure; TAPSE, tricuspid annular plane systolic excursion

bold font indicates $p < 0.05$
