# Supplementary material for: Left atrial area index provides the best prediction of atrial fibrillation in ischemic stroke patients: results from the LAETITIA observational study
Source: Front Neurol. 2023 Sep 27;14:1237550. doi: 10.3389/fneur.2023.1237550 (PMC10580428; doi:10.3389/fneur.2023.1237550)
Supplement: Supplementary file 3 [file Image_1.pdf]

Supplementary Figure

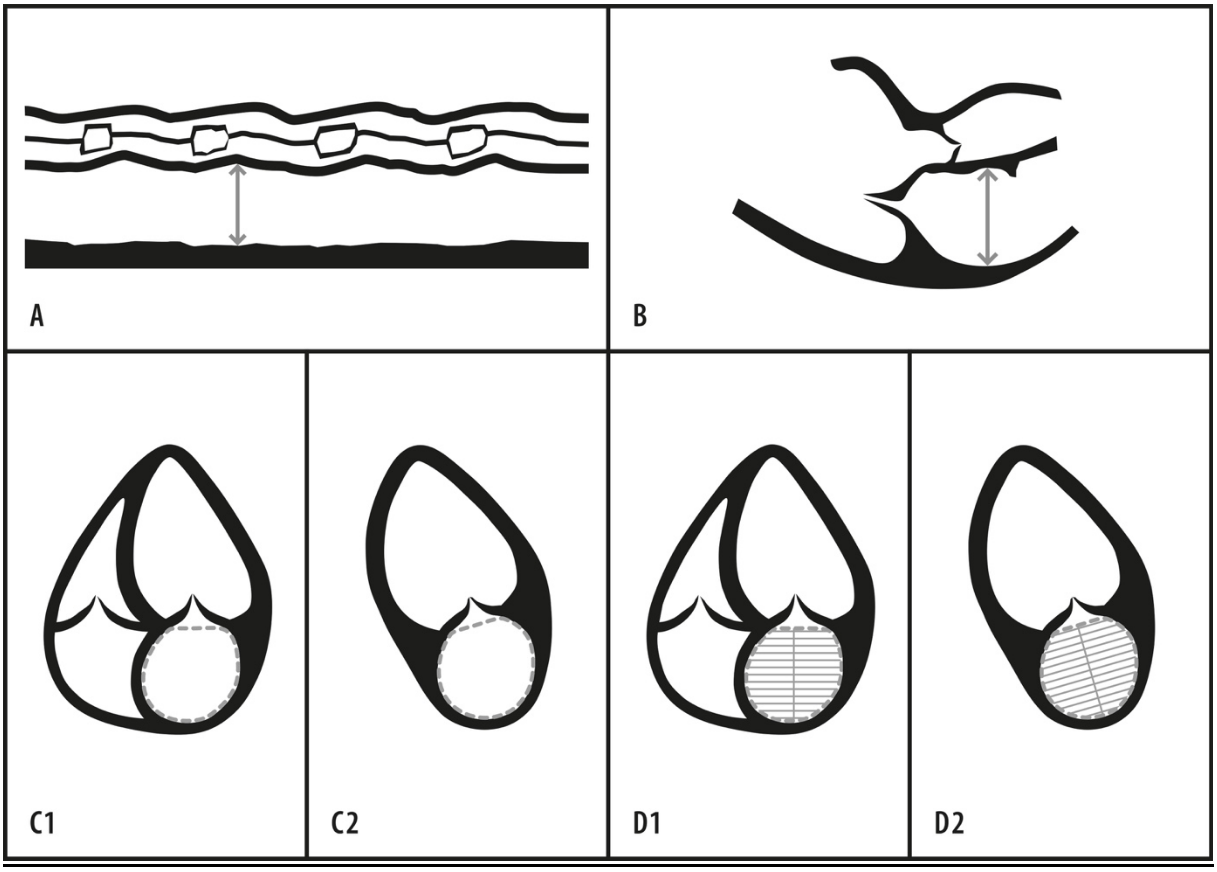

Determination of left atrial (LA) parameters by echocardiography. A) LA diameter in M-mode, B) LA diameter in two-dimensional parasternal long axis, C1) LA area in apical four-chamber and C2) two-chamber view, D1) LA volume in apical four-chamber and D2) two-chamber view.
